# Does the Chief Executive Officer (CEO)'s Educational Background Have an Impact on the Academic Hospital Leapfrog Safety Grades?

**DOI:** 10.7759/cureus.64033

**Published:** 2024-07-07

**Authors:** Daniel I Razick, Robert Monroe, Erik-Matthew Sario, Dana Aboukhalil, Navyaa Sinha, Vijay Khatri

**Affiliations:** 1 Surgery, California Northstate University College of Medicine, Elk Grove, USA

**Keywords:** education, ceo, leapfrog, quality, safety

## Abstract

Background

This study aims to investigate the potential association of the Chief Executive Officer (CEO)'s educational background on hospital safety metrics, specifically the Leapfrog safety grades.

Methods

A total of 172 academic and university-affiliated hospitals across the United States were included in the study. Information regarding a CEO's background was obtained through hospital websites, while the Leapfrog safety scores were found through the Leapfrog safety grade website. Hospital sizes were also noted.

Results

A total of 67 hospitals were led by Doctors of Medicine (MDs), 16 were led by Masters in Business Administration (MBAs), 14 were led by Masters in Healthcare Administration (MHAs), and 62 were led by CEOs with various other degree types. Of the 42 A-rated hospitals included, 17 were led by physicians and 25 by nonphysicians. The average Leapfrog grade point average was similar regardless of the CEO's background, ranging from 2.6 to 3.1.

Conclusion

The findings suggest that a CEO's educational background has less of an impact on the overall hospital safety performance than what may have previously been hypothesized.

## Introduction

The success of a healthcare organization is heavily influenced by the leadership at its helm, with the role of Chief Executive Officer (CEO) serving as a key component. The CEO's educational background and its impact on financial outcomes have been discussed in detail in several industries [1]. Previous studies have investigated the correlation between the CEO’s education and the operational outcomes of healthcare facilities [2]. However, there is a paucity of studies regarding the influence of the CEO's education on safety grading within the healthcare sector. Many healthcare organizations have physician leaders, while others do not. Hence, this study aims to comprehensively understand the significance of the CEO's education for patients and employees alike at academic hospital centers.

Current literature provides insights into the nuanced relationships between a CEO's educational background and its effect on healthcare institutional outcomes. However, there is no clear consensus on whether having a physician CEO compared to a nonphysician CEO impacts safety outcomes. A 2017 systematic review by Clay-Williams et al. sought to answer whether hospitals performed better with physician CEOs. It has been thought that physicians, with their clinical expertise, may prioritize safety measures, patient care, and the patient experience, while nonphysician CEOs may emphasize and implement financial strategies to maximize profits and minimize costs. However, the 2017 review was unable to make any definitive conclusions [3]. Therefore, there is certainly room for further exploration, particularly within academic institutions which must delicately balance educational goals and revenue targets, while providing excellent patient care. 

The significance of investigating the impact of the CEO's background on the Leapfrog safety grades cannot be overstated. The Leapfrog safety grades are powerful indicators of the ability of a hospital to provide safe and high-quality care and heavily influence public perception and trust in patient populations [4]. Leapfrog assigns a letter grade based on five categories: infections, problems with surgery, safety problems, practices to prevent errors, and doctors, nurses, and hospital staff. A letter grade of A is an indicator of excellent hospital safety, while an F indicates failure to meet safety standards. By analyzing academic hospital centers across the United States, the primary goal of this study is to elucidate the impact of the CEO's background, in particular physician versus nonphysician, on the Leapfrog safety grades. In doing so, we aim to better inform patients and providers alike, whether any significant differences are present between the CEO's educational types, which may impact overall hospital safety.

## Materials and methods

According to the HospitalView database, there are currently 216 hospitals considered to be academic medical centers [5]. However, for the year 2024, the Leapfrog safety grades were only available for 172 of these institutions. Therefore, 172 academic medical centers were included in the present study. Hospital centers were selected if both the Leapfrog safety grades and the CEO's background were publicly available from credible sources. This ensured a consistent and reliable dataset from credible sources.

The following data was extracted from the American Hospital Directory [6] and official hospital websites: CEO's name, CEO's educational background, and number of hospital beds. If the educational background of a CEO was unclear based on the hospital website description, a more detailed search was conducted utilizing social media platforms such as LinkedIn. The CEO's educational background was confirmed by cross-referencing a minimum of two independent sources. The Leapfrog safety grades and individual Leapfrog criteria scores (infection, problems with surgery, safety problems, practices to prevent errors, and doctors, nurses, and hospital staff) were extracted from the Leapfrog Hospital Safety Grade website [7]. The included hospitals were subsequently compared based on CEO type, the number of hospital beds, and Leapfrog safety grades. A one-way analysis of variance (ANOVA) was conducted to determine whether there was a statistically significant difference in the average grades of each CEO type. 

For the present study, the ethical board approval was deemed unnecessary as all information utilized was publicly available. All data were extracted from openly available sources such as the American Hospital Directory, official hospital websites, the Leapfrog Hospital Safety Grade website, and the CEO's public social media accounts. The research did not involve any private or sensitive information, patient data, or interventions and did not pose a risk to institutions or individuals. Therefore, the present study adhered to ethical standards without requiring formal board approval [8]. 

## Results

Breakdown of the CEO's background by degree type

**Figure 1:**
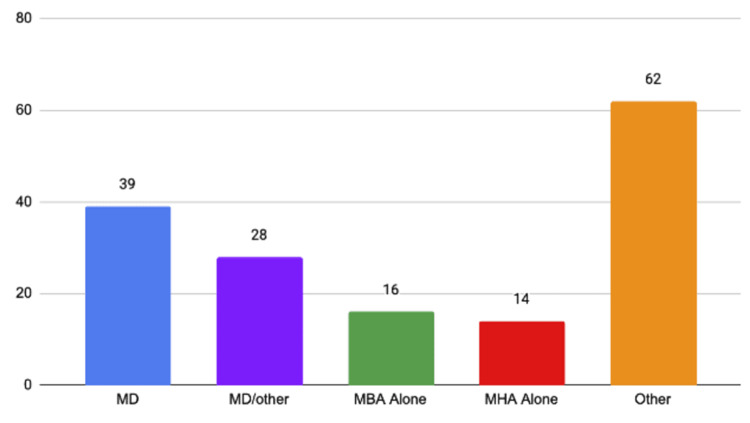
Breakdown of the CEO's background by degree type MD, Doctor of Medicine; MBA, Master of Business Administration; MHA, Master of Healthcare Administration

Across the 172 institutions surveyed, 159 CEOs were identified. Of the 159 analyzed, 39 held a Doctor of Medicine degree (MD), 28 held an MD and another degree (Masters in Business Administration (MBAs), Masters in Healthcare Administration (MHAs), etc.), 16 held an MBA alone, 14 held an MHA alone, and 62 held a combination of degrees including Doctor of Pharmacy (PharmD), Doctor of Philosophy (PhD), Juris Doctorate (JD), Masters in Mathematics, and Master in Health Services Administration. CEOs that held both an MHA and MBA made up the greatest proportion of the “other” category with six individuals. The exact breakdown of degrees held by the CEOs is summarized in Figure 1. 

Breakdown of MD degrees

**Figure 2:**
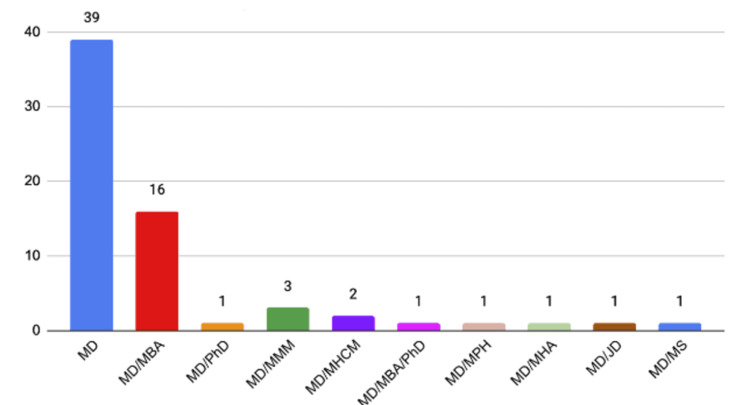
Breakdown of MD degrees MD, Doctor of Medicine; MBA, Master of Business Administration; MHA, Master of Healthcare Administration; PhD, Doctor of Philosophy; MMM, Master of Medical Management; MHCM, Master of Healthcare Management; MPH, Master of Public Health; JD, Juris Doctorate; MS, Master of Science

There were a total of 67 CEOs with medical degrees, 24% of which held MD/MBA degrees. Notably, only one CEO was a dual MD/JD. The exact breakdown of degrees held by physicians is summarized in Figure 2. 

Average number of hospital beds by CEO background

**Figure 3:**
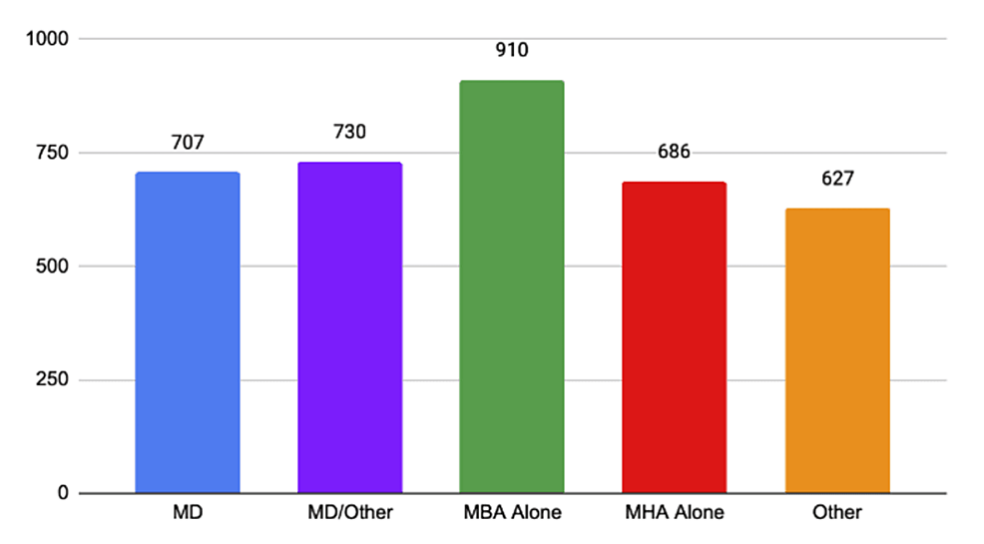
Average number of hospital beds by CEO background MD, Doctor of Medicine; MBA, Master of Business Administration; MHA, Master of Healthcare Administration

The average number of hospital beds has been included to better understand the size of the hospitals managed by each degree type (Figure 3). Subsequent tables and figures should be interpreted with this figure in mind. On average, MD-led hospitals contained 707 beds, hospitals led by MDs with another degree contained 730 beds, hospitals led by MBAs contained 910 beds, hospitals led by MHAs contained 686 beds, and hospitals led by a variety of degrees contained 627 beds. It should also be noted that there is significant variability in the number of hospitals managed by each degree type, and this may impact the results in Figure 3.

The Leapfrog safety grades and the CEO's educational background

Of the 172 institutions surveyed, the Leapfrog safety grades were available for 148. 42 were given a Leapfrog safety grade of A, 37 were given a grade of B, 57 given a grade of C, and 12 given a grade of D. Of the A-rated institutions, 17 were led by physician CEOs and 25 by nonphysician CEOs. Of the B-rated institutions, 18 were led by physician CEOs and 19 were led by nonphysician CEOs. Of the C-rated institutions, 20 were led by physician CEOs and 37 by nonphysician CEOs. Of the D-rated institutions, four were led by physician CEOs and eight by nonphysician CEOs. The exact breakdown of the Leapfrog grades is summarized in Table 1. 

**Table 1:** Leapfrog safety grades and CEO educational background MD, Doctor of Medicine; MBA, Master of Business Administration; MHA, Master of Healthcare Administration

	A	B	C	D	Total
MD	10	12	8	3	33
MD/Other	7	6	12	1	26
MBA	7	3	4	1	15
MHA	4	1	6	1	12
Other	14	15	27	6	62
Total	42	37	57	12	148

Leapfrog safety grade point averages by CEO educational background

**Figure 4:**
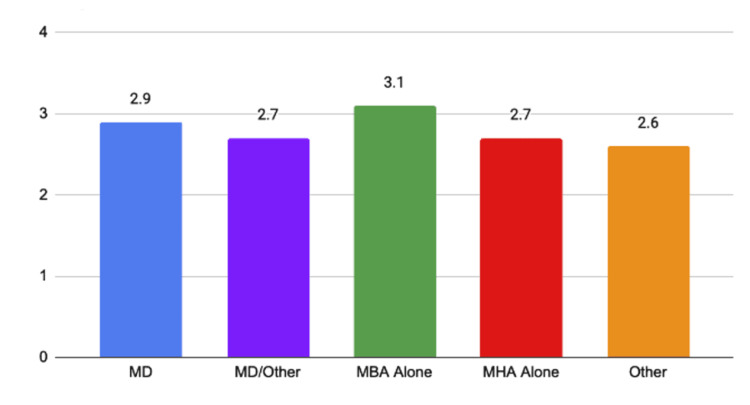
Leapfrog safety grade point averages by CEO educational background MD, Doctor of Medicine; MBA, Master of Business Administration; MHA, Master of Healthcare Administration

The grade point average (GPA) for CEOs of all types were similar ranging from 2.6 to 3.1. These numbers should be considered in the context of the number of hospitals led by each physician type as noted in Table 1 and the average number of hospital beds, an indicator of hospital size, as noted in Figure 3. A one-way ANOVA was conducted and found no significant difference in average grades (p-value = 0.413). Leapfrog GPAs by CEO type are summarized in Figure 4. 

## Discussion

Although some studies have found that the inclusion of physicians in the board of director positions for hospitals has reported positive associations with organizational performance [3], the present study’s examination of the Leapfrog Safety Rating shows that MD CEOs with no formal business education lead hospitals to nearly the same average safety standards as CEOs with no medical education whatsoever. Furthermore, when calculating the GPA for the Leapfrog Safety Ratings by CEO educational background as seen in Figure 4, we found no significant difference in any of the educational backgrounds. The lack of a statistically significant difference was confirmed via one-way ANOVA. Though this finding defies the intuition that medical education and experience in hospital leadership should improve the quality of care, there are a number of reasons it may not. Hospitals may be such large and complex organizations that the expertise of one individual is insufficient to make any medical difference. In fact, there is evidence that hospital CEOs have little to no ability to change the performance of their institutions one way or the other [9]. 

Our finding that CEOs who have an MD with an additional business degree fail to lead safer hospitals than either MD-only or business degree-only CEOs could indicate that a combination of these knowledge sets in a single individual will not intrinsically improve that individual's impact on the quality of that hospital, contrary to what might be expected. Alternatively, as the rigidly defined roles within medicine are relaxing and physicians are more freely able to move into management positions, the medical knowledge required to achieve high safety standards is available to the majority of CEOs via exposure to and consultation with physicians in management [10].

When examining the size of hospitals led by CEOs of different educational backgrounds in Figure 3, we found that each group led hospitals with similar bed counts, with the exception of those led by MBAs alone. This likely means that physicians are not limited to leading smaller hospitals than their business-educated counterparts. We postulate the potential implications of the sizes of hospitals led by each CEO type with regard to financial success. Physicians without formal business education may simply lack the skills and emphases required to make large institutions thrive financially. MD-only CEOs spend the majority of their lives developing their profession-specific skills and tend to apply a narrow vision and culture to their institutions [11]. Furthermore, these skills acquired either working alone or heading a small care team do not necessarily translate to the management of a large institution. CEOs are charged with leading several large teams, each composed of teams under them, while at the same time being responsible to a board of directors as well as to employees at every level of their institution. Physicians, by design as well as preference, work with a much greater degree of autonomy, being responsible primarily to their patients and direct supervisors. Additionally, there is an often overlooked distinction that must be made between the principles of leadership and those of management. Leadership is conceptually taught and emphasized to physicians in training and to a multitude of other professions and is typically concerned with visionary thinking among other things [12]. Overall, there are many factors that must be considered when analyzing the impact CEO education can have on hospital safety performance.

The scope of this study was primarily limited to the effect of a CEO's educational background on hospital safety performance and did not take into account other factors such as previous business experience and hospital geography. This study also did not analyze other factors that impact hospital safety grades including staffing, protocols, or budgets. Therefore, definitive conclusions cannot be made at this time regarding holistic safety of hospitals led by physician or nonphysician CEOs. Considering that a significant number of hospital CEOs hold an MD, it may also be worthwhile to investigate any potential impact medical specialty may have among institutions with physician CEOs.

## Conclusions

While physicians constitute a significant portion of academic hospital CEOs, there appear to be no statistically significant differences in hospital patient safety outcomes regardless of the CEO's educational background. The findings in the present study are not sufficient to conclude whether hospitals led by physician CEOs are safer than those led by nonphysician CEOs, as the Leapfrog safety grades are based on the collective performance of a hospital and are not a result of the educational background of one individual. Educational background, however, could potentially affect more specific aspects of hospital operation, such as adherence to the most recent clinical protocols, thus warranting further investigation.
